# Chemometrics Approach Based on Wavelet Transforms for the Estimation of Monomer Concentrations from FTIR Spectra

**DOI:** 10.1021/acsomega.3c01515

**Published:** 2023-05-23

**Authors:** Araki Wakiuchi, Swarit Jasial, Shigehito Asano, Ryo Hashizume, Miho Hatanaka, Yu-ya Ohnishi, Takamitsu Matsubara, Hiroharu Ajiro, Tetsunori Sugawara, Mikiya Fujii, Tomoyuki Miyao

**Affiliations:** † Materials Informatics Initiative, RD technology and digital transformation center, JSR Corporation, 3-103-9 Tonomachi, Kawasaki-ku, Kawasaki, Kanagawa 210-0821, Japan; ‡ Data Science Center, Nara Institute of Science and Technology, 8916-5 Takayama-cho, Ikoma, Nara 630-0192, Japan; § Graduate School of Science and Technology, Nara Institute of Science and Technology, 8916-5 Takayama-cho, Ikoma, Nara 630-0192, Japan; ∥ JSR Corporation Yokkaichi Research Center, 100 Kawajiri-cho, Yokkaichi, Mie 510-8552, Japan; ⊥ Department of Chemistry, Faculty of Science and Technology, Keio University, 3-14-1 Hiyoshi, Kohoku-ku, Yokohama, Kanagawa 223-8522, Japan

## Abstract

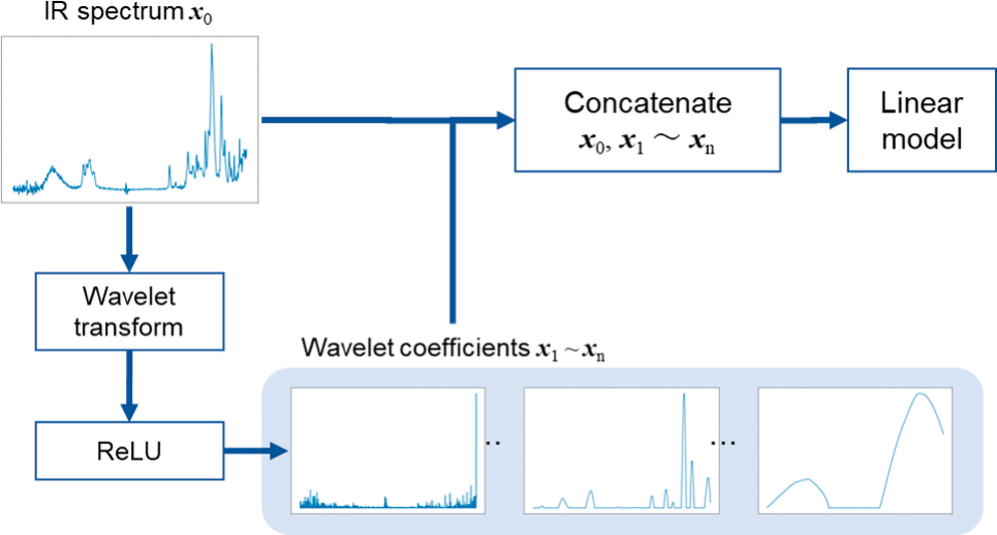

Fourier-transform infrared (FTIR) spectroscopy can detect the presence of functional groups and molecules directly from a mixed solution of organic molecules. Although it is quite useful to monitor chemical reactions, quantitative analysis of FTIR spectra becomes difficult when various peaks of different widths overlap. To overcome this difficulty, we propose a chemometrics approach to accurately predict the concentration of components in chemical reactions, yet interpretable by humans. The proposed method first decomposes a spectrum into peaks with various widths by the wavelet transform. Subsequently, a sparse linear regression model is built using the wavelet coefficients. Models by the method are interpretable using the regression coefficients shown on Gaussian distributions with various widths. The interpretation is expected to reveal the relation of broad regions in spectra to the model prediction. In this study, we conducted the prediction of monomer concentration in copolymerization reactions of five monomers against methyl methacrylate by various chemometric approaches including conventional methods. A rigorous validation scheme revealed that the proposed method overall showed better predictive ability than various linear and non-linear regression methods. The visualization results were consistent with the interpretation obtained by another chemometric approach and qualitative evaluation. The proposed method is found to be useful for calculating the concentrations of monomers in copolymerization reactions and for the interpretation of spectra.

## Introduction

Calculating the concentrations of reactants is an established approach to monitor chemical reactions. For this purpose, various instruments and methods can be employed, such as nuclear magnetic resonance (NMR), infrared (IR), near-infrared (NIR), and ultraviolet–visible light (UV–vis) spectroscopies.^1−5^ Some methods require isolation techniques using polar columns, non-polar columns, size exclusion columns, filtration, centrifugation, and liquid–liquid extraction before analysis, which hinders rapid and efficient identification of the concentrations.

Fourier-transform infrared (FTIR) spectroscopy can rapidly obtain an infrared spectrum of a substance via the Fourier transform of interferential light. FTIR spectra convey information about the normal modes of vibration in a molecule that helps in the identification of the functional groups present. Furthermore, since the absorbances of a solution at structure-specific wavenumbers are proportional to the concentration of the solution, i.e., the Beer–Lambert law, it can be used for calculating the concentrations of reactants. However, in practical applications, concentration estimation by FTIR is generally difficult because the peaks of absorbance from different components are usually overlapped, and their intensities are not necessarily proportional to the concentrations of components due to other interactions, e.g., hydrogen bonding. In this study, we focus on copolymerization reactions, where various peaks of comonomers and polymers are overlapped. Some functional groups present in comonomer molecules also remain in polymer molecules. Atomic environments vary from one reaction to another due to different reaction conditions, and wavenumbers for the disappearing double bonds of comonomers are expected to be overlapped due to their structural similarity. The estimation of the concentrations of reactants is expected to be more difficult than that of a mixture without reaction.

Analyzing IR and NIR spectra to obtain quantitative information about the substance is a fundamental topic in the field of chemometrics.^6−9^ Various methods have been proposed for this purpose, mainly using linear regression (classification) models and dimensionality reduction techniques.^10−17^ For example, Arakawa et al. proposed a genetic algorithm-based wavelength selection (GAWLS) in combination with partial least squares regression (PLS). They applied the method to two different objectives: the soluble solid content of apples and the concentrations of moisture, nitrogen, and carbon in soil.^18^ Since these techniques have been applied to mixtures measured under controlled conditions, it is uncertain whether or not these methods can be useful for estimating monomer concentrations during polymerization reactions, where various peaks are intertwined.

Decomposing peaks with different widths and locations from a spectrum can be achieved by wavelet transforms (WTs). The WT converts a spectrum into the trends of constituent peaks with different widths. The WT has been utilized for chemometric analysis.^19,20^ In particular, it has also been utilized for IR spectrum analyses for compound identification,^21^ noise reduction,^22^ and classification of biological samples in a combination of machine learning (ML) models.^23−27^

Herein, we propose a chemometric method of estimating the concentrations of monomers from a solution of polymerization reaction by utilizing WT. The input solution spectrum is converted to the WT coefficients (features). These coefficients are put into a robust and sparse linear regression model generated by the elastic net (EN) to predict the concentration. Furthermore, models in the proposed method are interpretable based on the regression coefficients by projecting them on approximated wavelets on the input spectrum. We prepared the data sets of FTIR spectra of the copolymerization reactions consisting of six different monomers. Our proposed workflow showed better predictive performance than other methods, including GAWLS and various non-linear and linear ML algorithms. Furthermore, we made the data sets and our implementation of the WT features-based prediction method available in the public domain (https://github.com/Wa-Araki/WT-ENCV), which we hope will contribute to further development in this field.

## Materials and Methods

### Free-Radical Copolymerization

**Figure 1:**
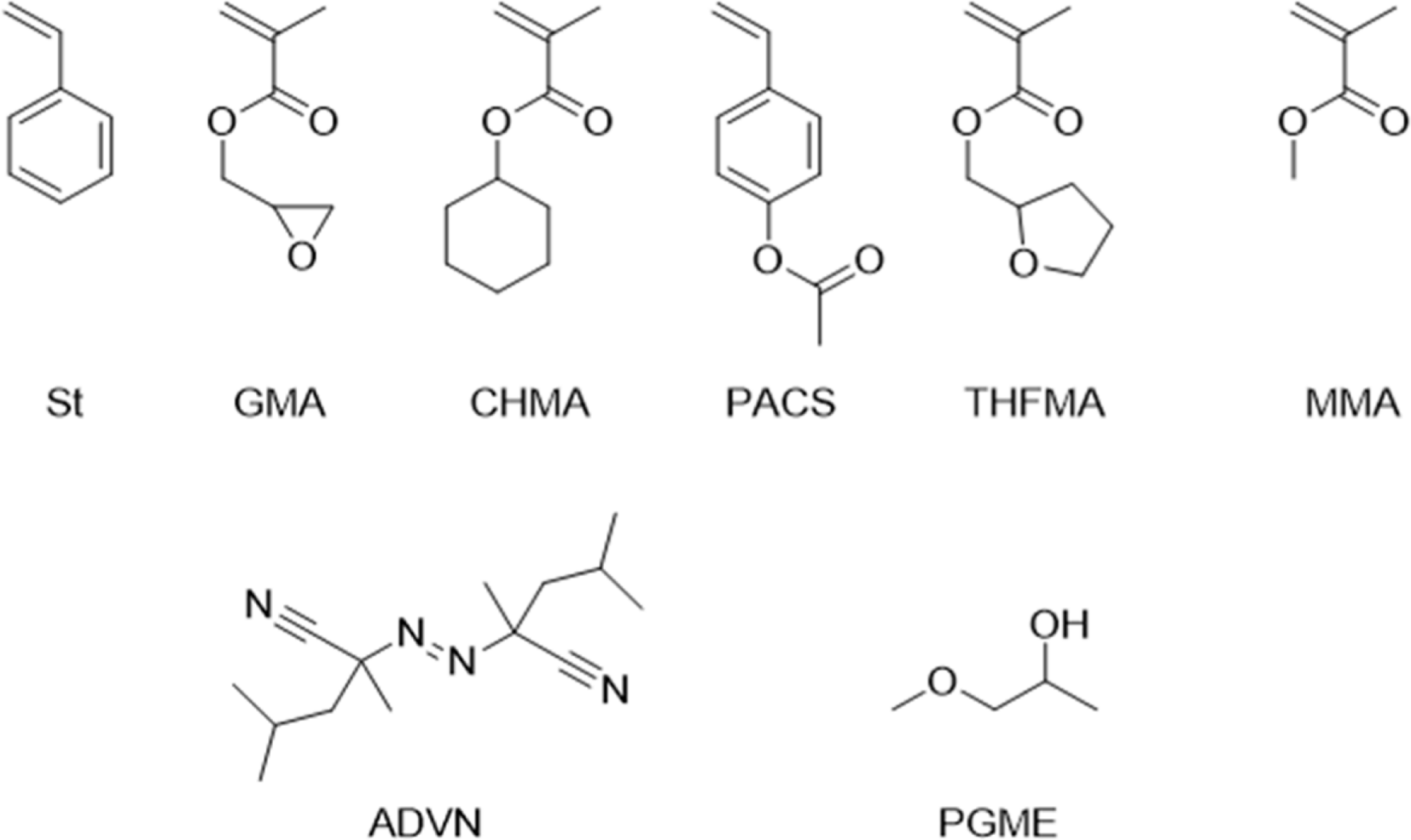
Compounds in polymerization reactions. The chemical structures of the six monomers are shown in the upper row; St: styrene, GMA: glycidyl methacrylate, CHMA: cyclohexyl methacrylate, PACS: *p*-acetoxystyrene, THFMA: tetrahydrofurfuryl methacrylate, and methyl methacrylate. In the lower row, the chemical structures of the initiator of ADVN: 2,2′-azobis-2,4-dimethylvaleronitrile and the solvent PGME: propylene glycol methyl ether are shown.

Free-radical copolymerization is a polymerization method where repeating units with radicals are successively added to a polymer chain. In this study, six monomers were used: styrene (St, FUJIFILM Wako, special grade), glycidyl methacrylate (GMA, FUJIFILM Wako, 1st grade), cyclohexyl methacrylate (CHMA, TCI), *p*-acetoxystyrene (PACS, Tosoh Finechem), tetrahydrofurfuryl methacrylate (THFMA, FUJIFILM Wako, 1st grade), and methyl methacrylate (MMA, FUJIFILM Wako, special grade). Each of the first five monomers reacts with MMA. In all reactions, propylene glycol monomethyl ether (PGME) was used as a solvent and 2,2′-azobis(2,4-dimethylvaleronitrile) (ADVN, FUJIFILM Wako, 1st grade) as a thermal initiator. Figure 1 shows their structural formulas.

In copolymerization reactions, we dissolved St, GMA, CHMA, PACS, or THFMA with MMA and ADVN in PGME and bubbled the solution by nitrogen gas for 3 min. The solution was heated in a silicone oil bath and sampled at certain intervals for component analysis.

### IR Spectrum Measurement

We used FTIR-6800 (JASCO) to measure IR spectra. To improve measurement efficiency, a measurement method was applied to acquire absorbance at multiple wavelengths at once by continuously irradiating infrared light, followed by Fourier transforming the interference patterns caused by the sample. For each sample, a total of 32 cumulative scans were taken in the attenuated total reflection mode with a resolution of 1 cm^–1^ and a frequency range of 4000 to 600 cm^–1^.

### Monomer Concentration Determination

High-performance liquid chromatography (HPLC) with photo diode array (PDA) detectors was used to quantify the monomer concentrations. The HPLC equipment by Shimadzu Corporation consisted of a degasser (DGU-20A3), a liquid chromatograph (LC-20AB), an auto sampler (SIL-20A), a PDA (SPD-M20A), a column oven (CTO-20A), a guard column (Inertsil ODS-3 5 μm 4.0 × 250 mm), and a reversed-phase column (Inertsil ODS-3 5 μm 4.6 × 250 mm). To separate MMA and the other monomer in each reaction solution and to measure their concentrations, a 55:45 mixture of acetonitrile and distilled water was used at 1.0 mL/min at 40 °C.

### Data Sets for Concentration Prediction Models

For five comonomer pairs, monomer concentrations in solutions of the free-radical copolymerization were measured along with the corresponding IR spectra. One component of every monomer pair was fixed to MMA; thus, five reaction types were tested, forming five data sets. For each data set, IR spectra became independent variables, with concentration being the objective variable for ML. Table 1 reports statistics of the data sets. Overall, the averages of monomer concentrations were similar irrespective of reaction types. For St, 128 samples were measured, while for CHMA, only 28 samples were evaluated. The data sets used in this study are available (Dataset.xlsx) in the Supporting Information.

**Table 1:** Data Set Profiles^a^

		concentration [mmol/g]		
monomer	no. of samples	mean (sd^b^)	maximum	minimum
St	128	0.67 (0.35)	1.44	0.09
GMA	62	0.42 (0.19)	0.94	0.11
CHMA	28	0.46 (0.19)	0.93	0.19
PACS	29	0.60 (0.25)	1.10	0.21
THFMA	58	0.47 (0.23)	1.20	0.15

a For each copolymerization reaction, monomers are listed in the first column. The other monomer was MMA for the all reactions.

b Standard deviation.

### Proposed Method for Monomer Concentration Prediction

**Figure 2:**
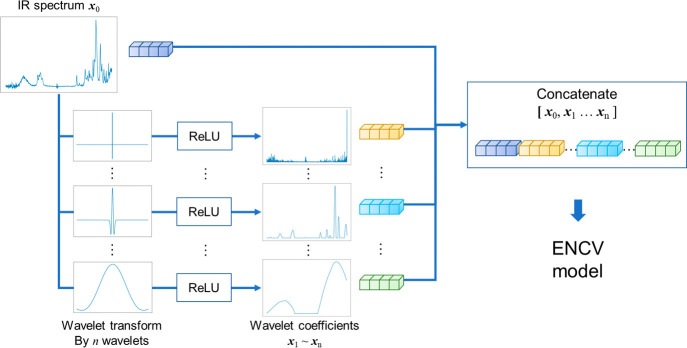
Concept of the elastic net optimized by CV using wavelet-transformed features (WT-ENCV).

For predicting monomer concentration from an IR spectrum, we propose to use EN regression models and WT features as model inputs. Furthermore, a direct visualization method for model interpretation based on regression coefficients is proposed. The proposed method is termed as EN optimized by cross-validation (CV) using WT features (WT-ENCV). The input of the model is a concatenated vector of the original IR spectrum and WT vectors. The hyperparameters of the EN model are tuned by CV. The overview of WT-ENCV is provided in Figure 2. Wavelet coefficients are applied to rectified linear units (ReLU) to generate positive WT features. A detailed explanation of WT, EN, and the visualization technique is provided as follows. In the following, WT-ENCV, where the number of types of wavelet functions used is *n*, is denoted as WT*n*-ENCV.

#### Wavelet Transform

As the wavelet function, the Ricker wavelet was employed in this study. It is defined as follows (parameterized by *w* for *x*)
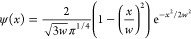
where *w* is a parameter corresponding to the width of a wavelet function. In this study, a set of *w* values corresponding to *n* wavelets were employed, as determined by the following equation
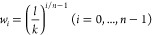
where *l* is the length of IR spectrum data, *n* is the number of wavelets, and *k* is a parameter to control how wide the widest wavelet is in relation to *l*. Each spectrum was binned into a vector of 3257, whose interval was approximately 1 cm^–1^. In this study, the value of *k* was set to 4 because the Ricker function should lie within the range of spectra. The corresponding *w* values ranged from 1 to 881.75. A wavelet coefficient could be calculated for each point of an input spectrum by the convolutional operation between the wavelet and the spectrum. All WT calculations were carried out using SciPy (version 1.7.3).

#### Elastic Net

EN is a regularized multivariate linear regression method, where the following loss function is minimized
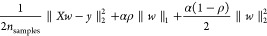
where *X* is a set of independent variables for samples (design matrix), *y* is a vector of objective variable values for the samples, *w* is a coefficient vector, and α and ρ are the hyperparameters to control the strength and balance of the *l*_1_ and *l*_2_-norm regularization terms, respectively.^28^ In ENCV, these hyperparameters were optimized by five-fold CV. When scaling the input *X* as a pre-processing step, an individual scaling operation was employed so that each feature in the training set fell within the range of 0 to 1 (min–max normalization). All the calculations for the scaling and EN models were carried out using Scikit-learn (version 0.24.2).

#### Visualization of Regression Coefficients

Since the ENCV model is a linear regression model, the regression coefficients for WT coefficients provide an interpretation of the model. These coefficients can be projected on a set of Ricker functions parameterized by multiple *w*s, corresponding to different widths of peaks.

Our proposed method focuses on the visualization of the regression coefficients on a set of Gaussian functions that approximate the Ricker functions. The Gaussian functions are placed on the centers (positions) of the Ricker functions with their corresponding widths. These Gaussian functions are further scaled based on the coefficients. It should be noted that although projecting regression coefficients on the original Ricker functions is possible, the visualization results became too complicated due to the overlap among the Ricker functions. Thus, we decided to use Gaussian functions instead of the original wavelet functions in this study.

**Figure 3:**
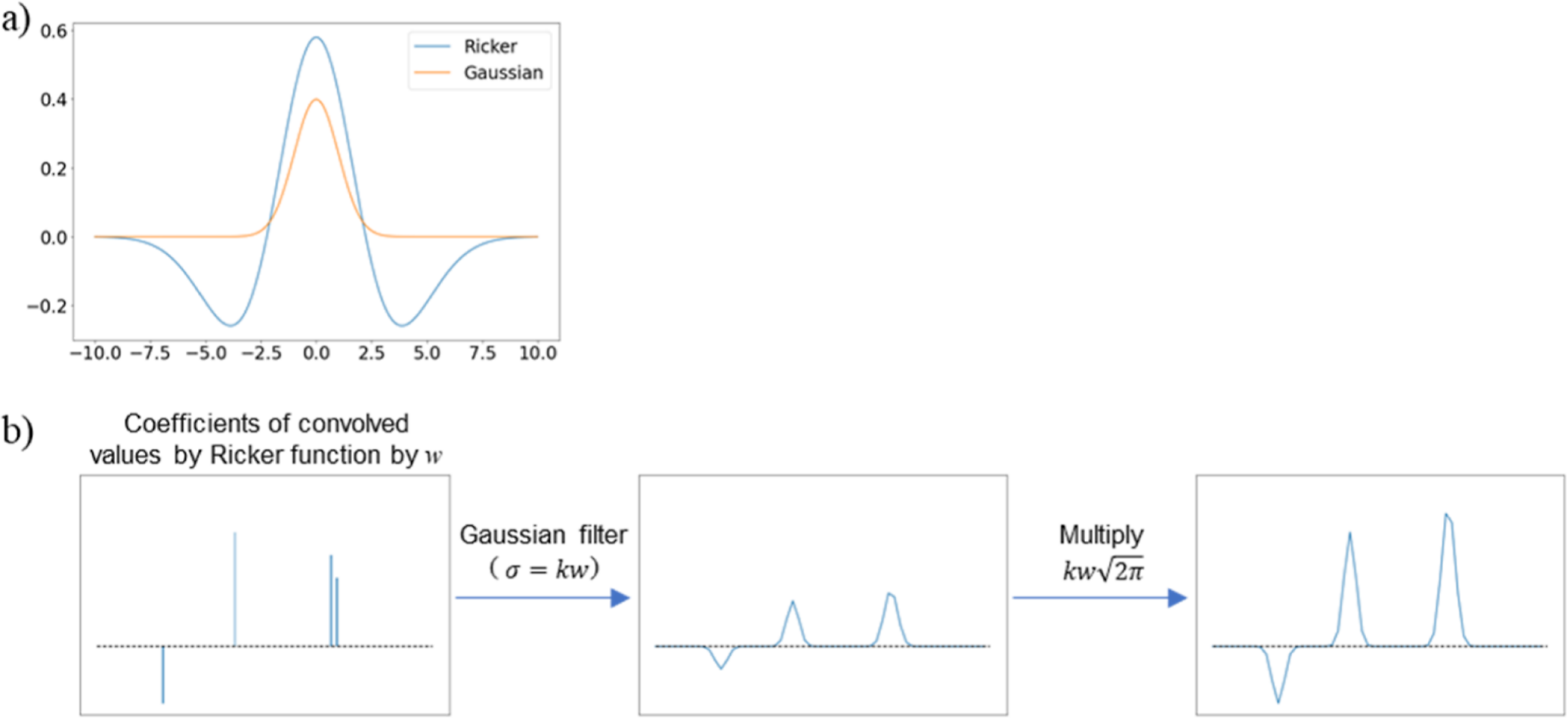
Visualization procedure. Ricker function approximated by the Gaussian function with a σ value of 0.45 (a). Projection of a set of coefficients for Ricker functions with the same *w* value (b).

The mean (center) of each Gaussian function is equal to that of the corresponding Ricker function. σ, which determines the width of the Gaussian function, was determined by finding the ratio of *w* to the maximum value of the Gaussian function convolved by the Ricker function (Figure 3a). Our pre-analysis revealed that a value of 0.45 for the σ/*w* well approximates the Ricker function parameterized by *w*. Therefore, in this study, σ was set to 0.45*w*. Then, the Gaussian functions were rescaled by multiplying the regression coefficients and  (Figure 3b).

### ML Algorithms for Comparison

For comparison, well-established ML algorithms for spectrum analyses were also tested including automatic relevance determination regression (ARD),^29^ PLS,^16^ GAWLS,^18^ regression models combined with recursive feature elimination (RFE),^30^ support vector regression (SVR),^31^ random forest (RF),^32^ and LightGBM.^33^

#### Bayesian Automatic Relevance Determination Regression

ARD is a Bayesian linear regression method that assumes a Gaussian prior to regression coefficients. In ARD, the precision of each coefficient is represented as a γ prior, and a sparse model (many coefficients tend to be zero) is produced after fitting to data. One of the advantages of ARD is that no hyperparameters need to be optimized due to its Bayesian learning. A threshold value for pruning the weights with high precision from computation (threshold λ) was set to 10.

#### PLS Regression

PLS regression identifies a small set of latent variables (components) that exhibit the maximum covariance between the latent and objective variables for a regression model. This method aims to make a stable regression model even when the number of independent variables is larger than the number of samples and when collinearity among independent variables exists. The number of components in PLS was chosen from the range 1 to 20 by five-fold CV using a training data set.

#### Genetic Algorithm-Based Wavelength Selection

GAWLS refers to PLS regression using selected variables by the genetic algorithm (GA). Optimal variables are selected as a set of continuous regions of wavelengths, leading to a clear interpretation of PLS models. GAWLS was reported for several applications and exhibited easy-to-interpret results. In this study, GA population was set to 100; the number of GA generations used was 15, the number of areas or wavelength regions selected was 5, cross-over and mutation probability were 0.5 and 0.2, respectively, the maximum number of components of PLS was set to 10, and the number of PLS CV folds was 5.

#### Support Vector Regression

SVR is a regression method based on support vector machines. It can be employed as a nonlinear regression model by introducing a kernel function. SVR is used as a robust regression method by introducing the ε-insensitive hinge loss combined with the *l*_2_-norm regularization term. In this study, the radial basis function (RBF) was used as a kernel function. The hyperparameters: *C*, ε, and γ in RBF were optimized using five-fold CV on a training data set.

#### RF Regressor

RF is an ensemble regression method of decision trees, where many decision trees are generated with different sets of samples or variables. The output of RF is the average of the outputs of the trees. Ensemble learning can prevent over-training. In this study, the number of trees was set to 100 (default number in the Scikit-learn API), and the minimum number of samples required to reach the leaf node (*min_samples_leaf*) was optimized by five-fold CV.

#### LightGBM Regressor

LightGBM is a gradient-boosting decision tree method characterized by its efficiency and scalability for handling large, high-dimensional data. The two key techniques gradient-based one-side sampling and exclusive feature bundling help reduce data scan time and improve overall performance. In this study, a set of hyperparameters (*learning_rate*, *n_estimators*, *num_leaves*, *boosting_type*, *min_child_weight*, *min_child_samples,* and *colsample_bytree*) in the *lightgbm* library was optimized by five-fold CV.

#### Recursive Feature Elimination (Variable Selection Method)

RFE is a backward stepwise feature selection method combined with a regression model. In each step of RFE, the weakest independent variable is eliminated until the number of variables reaches a specified number. The least important variable is the one exhibiting the smallest absolute regression coefficient. In this study, RFE combined with ENCV and linear SVR was used, and the limit of the number of features was set to 10. In each iteration, 10 features were eliminated from the remaining features.

### Conventional Approach to Concentration Estimation

Following the traditional/conventional method of concentration estimation, pure spectra of five monomers—St, GMA, CHMA, PACS, and THFMA—were obtained, and peaks were extracted for each monomer from their respective spectra. The peaks were selected using the peak-finding algorithm from the SciPy module of Python (*signal.find_peaks*), where *prominence*, which is the minimum height necessary to descend to get from the summit to any higher terrain, was used as a parameter for selection (*prominence* = 0.06). Peak regions which were overlapping with the peaks in pure spectra of counterpart monomer MMA and solvent PGME, within the overlap range of [peak + 10, peak – 10], were removed to solely focus on individual monomer peaks. A correlation between absorbance and residual monomer concentrations was calculated for each monomer. Linear regression was conducted by taking absorption spectra at the selected peaks as independent variables and the residual concentration of individual monomers as the dependent variable. Linear regression analysis was conducted individually on the selected peaks as well as a combination of peak regions for each monomer.

### Software and Implementation

Models with ARD, PLS, RFE with ENCV, and linear SVR, SVR, and RF were implemented in Python scripts using Scikit-learn (version 0.24.2) libraries. For CV, the GridSearchCV class of Scikit-learn was used. For LightGBM, lightgbm (version 3.3.5) was used. For GAWLS, an in-house implementation by a Python script was used.

### Validation Procedure

For each data set of a monomer pair, a five-fold CV validation was repeated 5 times by changing the seed of the random number generator, meaning different training/test splitting. In each CV validation, monomer concentrations were iteratively predicted for a fold-out test data set by the models trained and optimized only on the fold-in data sets. Using the predicted and ground-truth monomer concentrations, performance evaluation metrics were calculated. The calculated metric was the coefficient of determination (*R*^2^). For each data set, average metric values over the five trials are reported as predictive ability.

## Results and Discussion

### Global Prediction Performance

For the data sets of five monomer combinations, *R*^2^ scores of the 5 times CV’s prediction results with various prediction models are reported in Table 2. Tested prediction methods were ENCV, WT10-ENCV, ARD, PLS, GAWLS, RFE-ENCV, RFE-SVR, RF, SVR, and LightGBM. For one CV trial (seed 0), method-wise predicted vs observed concentration plots for each monomer combination are also reported in Figures S10–S14. Overall, the predictive ability of linear regression models was higher than that of non-linear ones. This result was also supported by the theoretical background, i.e., Beer’s law, and also by an empirical knowledge that linear models are preferable for chemometric analysis on IR or NIR spectra. Among the linear modeling methods, the proposed WT10-ENCV performed better than others: for St, PACS, and THFMA, it performed the best, and for GMA and CHMA, *R*^2^ values were close to 0.9 (0.88 and 0.87, respectively). For St and THFMA, WT10-ENCV was statistically better than the second-best method at the significance level of 0.05, including GAWLS.

**Table 2:** Predictive Performances^a^^,^^b^

	linear models							nonlinear models		
	**WT10-ENCV**	ENCV	ARD	PLS	GAWLS	RFE-ENCV	RFE-SVR	RF	SVR (RBF)	LightGBM
St	0.95*	0.91	0.91	0.92	0.90	0.93	0.92	0.87	0.88	0.91
GMA	0.88	0.79	0.89	0.71	0.81	0.81	**0.90**	0.42	0.62	0.41
CHMA	0.87	0.86	0.94*	0.71	0.78	0.86	0.89	0.39	0.40	0.24
PACS	**0.89**	0.87	0.86	0.84	0.78	0.82	0.86	0.81	0.60	0.32
THFMA	0.80*	0.72	0.72	0.61	0.65	0.66	0.71	0.50	0.40	0.49

a For each data set and prediction model, the average of five *R*^2^ values for the five-fold CV is reported. Asterisk marks show that the models are statistically better than the second-best method at the significance level of 0.05.

b ENCV: elastic net with hyperparameters determined by cross-validation, WT10-ENCV: elastic net with wavelet-transformed features, ARD: Bayesian automatic relevance determination regression, PLS: partial least squares regression, GAWLS: genetic algorithm-based wavelength selection, RFE: recursive feature elimination, SVR: support vector regression, RF: random forest, RBF: radial basis function, and LightGBM: light gradient boosting machine.

As a control, the conventional method of concentration identification was applied using the pure spectra of individual monomers (see the Materials and Methods section). The resulting averaged *R*^2^ values were very small for individual peaks (Table S1) and also for a combination of peaks (Table S2), except for PACS. Even for PACS, the average *R*^2^ value of 0.82 is smaller than that from ML models trained without prior knowledge of monomer peaks (Table 2). Most of the peaks that obtained high *R*^2^ were the peaks with the highest correlation to residual M1 concentration (Table S1). From the above experiments, linear regression models solely using the reaction spectral data set performed better for the concentration prediction of monomer components, in particular WT10-ENCV worked superior to other approaches.

### Effect of Hyperparameters in WT-ENCV on Prediction Performance

Overall, the proposed WT-ENCV worked better when the number *n* was 10 for the parameterized wavelets. Table 3 shows *R*^2^ results for ENCV, WT10-ENCV, WT20-ENCV, and WT30-ENCV using the spectral data sets. Furthermore, the effect of scaling was investigated when combined with WT10-ENCV [WT10-ENCV (scaling) in Table 3]. The tested scaling method for the spectrum was range scaling. The prediction performance was found to be similar with/without scaling for *n* = 10. However, introducing WT did improve the model performance as shown in comparison to the column of ENCV in Table 3.

**Table 3:** Predictive Performances for Different *k* Parameters in WT-ENCVs^a^

	ENCV	WT10-ENCV	WT20-ENCV	WT30-ENCV	WT10-ENCV (scaling)
number of wavelet functions *n*	0	10	20	30	10
number of coefficients	≤3527	≤38,797	≤74,067	≤109,337	≤38,797
St	0.91	**0.95**	**0.95**	**0.95**	**0.95**
GMA	0.79	**0.88**	0.87	**0.88**	**0.88**
CHMA	0.86	0.87	0.86	0.87	**0.90**
PACS	0.87	**0.89**	0.85	0.86	0.87
THFMA	0.72	**0.80**	0.79	0.79	0.75

a For each data set and prediction model, the average of five *R*^2^ values for the five-fold CV is reported.

### Linear Regression Models Using WT Features

In theory, WT features can be combined with any ML models. The proposed interpretation method can be applied only when WT features are combined with a linear regression model. ARD, PLS, and RFE-SVR were further tested using the WT10 features. For each combination of a data set and a linear regression model, the average of five *R*^2^ values for the five-fold CV is reported in Table 4. For RFE-SVRCV using WT10 features, 3000 features were eliminated per iteration until 3527 features remained (number in original IR spectra), and 10 features were eliminated per iteration from the remaining features to select a set of 10 best features. Overall, ENCV models worked better than the other linear regression models, followed by RFE-SVR. However, the calculation time for ENCV was much shorter than for RFE-SVR. Thus, ENCV was a good linear regression model for the large number of WT features (Table 3), automatically reducing variables during training the model.

**Table 4:** Predictive Performances for Linear Models Using WT10 Features^a^

	ENCV	ARD	PLS	RFE-SVR
St	0.95*	–1.55	0.72	0.92
GMA	0.88	0.81	0.36	0.89
CHMA	0.87	0.90	0.46	0.84
PACS	0.89	0.70	0.65	0.83
THFMA	0.80*	0.08	0.48	0.75

a For each data set and prediction model, the average of five *R*^2^ values for the five-fold CV is reported.

### Interpretation of WT-ENCV Models by Coefficient Visualization

The number of variables in a WT-ENCV model is significantly larger than the original spectrum length (Table 3). However, this number is drastically reduced when combined with sparse linear regression techniques. Each regression coefficient corresponds to the single wavelet centered on a wavenumber. We propose to visualize this information using a set of Gaussian functions that well approximate the corresponding Ricker functions (see Materials and Methods).

**Figure 4:**
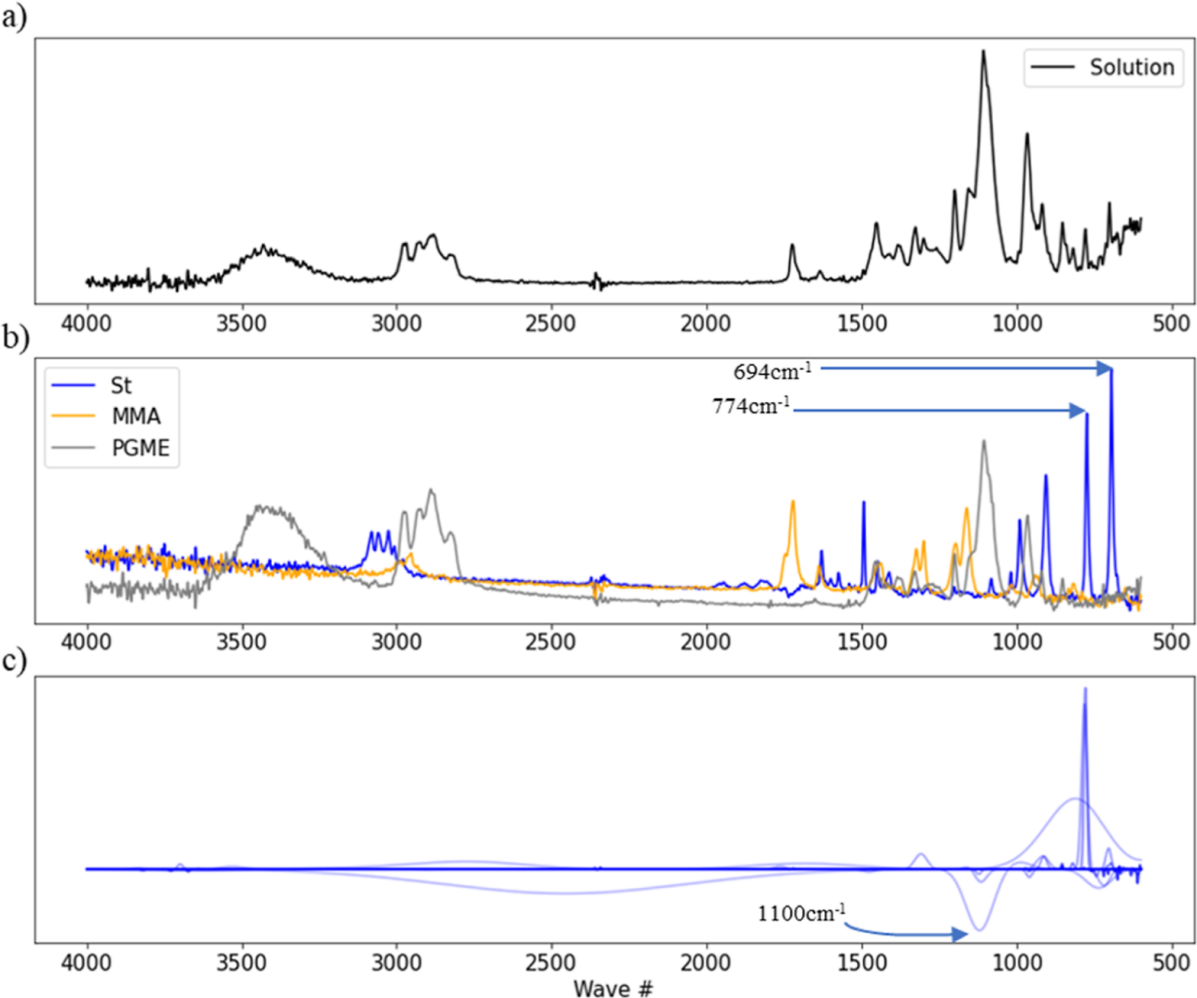
Interpretation of a WT10-ENCV model for a St concentration prediction. Spectrum of B-5r1_1 solution for St (0.87 mmol/g) and MMA (0.80 mmol/g) as an example (a), FTIR spectra of monomers and PGME (b), and the visualization of the contribution of peaks for the predicted concentration from the average of coefficients in all trained WT10-ENCVs (scaling) obtained from 5 times of five-fold CV (c) are shown.

Figure 4 shows an example visualization output for the St concentration prediction by WT10-ENCV with scaling. When only focusing on peaks in the St monomer spectrum, both peaks at 774 and 694 cm^–1^ seemed influential to the calculation of the concentration of St (Figure 4b). However, the visualization result in Figure 4c clearly indicates that the sharp peak around 774 cm^–1^ is more valuable than around 694 cm^–1^. This finding is also supported by comparing with the solution peaks (Figure 4a). In the region below 600 cm^–1^ in this spectrum, baseline variation derived from PGME has a significant effect, indicating the difficulty of making quantitative assessments based on the peak area in this region. The results were also compared with important wave numbers/regions selected from GAWLS as a control method. For GAWLS models, the most important wave numbers/regions were ranked based on their frequency of occurrence over all the models in CV. As shown in Figure S5, the wave region around 777 cm^–1^ was selected by GAWLS, which is in close proximity with the results from WT10-ENCV models. Hence, the interpretation was found to be similar. By the proposed method, important peaks with widths can be automatically extracted without analyzing peaks of all the components in the solution.

The broad negative contribution was observed around 1100 cm^–1^ in Figure 4c. Several peaks were observed for MMA and one large, broad peak for PGME. On the other hand, no St-derived peaks were detected. This implied that the information at this position is utilized to subtract the influence of components other than St. The above observation suggests that the absorbance around 1100 cm^–1^ is used to subtract unnecessary area values in determining the density of St. Taken together, our visualization approach to the regression coefficients helps to understand the prediction models of monomer concentrations after considering both peak places (wavenumbers) and the width of peaks.

## Conclusions

FTIR spectroscopy can rapidly obtain an infrared spectrum directly from a mixture. It can be utilized for monitoring the concentrations of components in chemical reactions. In this study, we propose a chemometrics approach to predict the concentration of components from a FTIR spectrum. The proposed method first transforms a spectrum to a set of wavelet coefficients, followed by modeling using a sparse linear regression algorithm of the elastic net. In addition, the model is interpretable by assigning correlation coefficients on the corresponding Gaussian functions with various widths. Five different types of copolymerization reactions against MMA were prepared, and spectrum data for modeling was accumulated. When evaluating the various regression models by five-fold CV 5 times, the proposed method statistically outperformed conventional methods for two of the five monomer combinations and showed overall stable predictive performance. Furthermore, the visualization of the correlation coefficients on the Gaussian functions directly led to the interpretation of the models in terms of broad regions of spectra, which are supported by the important regions selected by GAWLS and by the interpretation by researchers.

Thus far, we have only focused on FTIR spectra in solutions of copolymerization reactions. However, we believe that the proposed method will also be applicable not only to the FTIR spectra of other materials but also to spectra obtained from different spectroscopies. For example, in UV–vis, the quantification of specific structures is often calculated just based on the intensity of peak tops. Also, NMR spectrum analysis for polymers might be a good application because NMR peaks tend to be broadened by the inhibition of the polymer-free movement.

## Supporting Information Available

Visualization results by WT10-ENCV for THFMA, CHMA, GMA, and PACS; selected wavenumbers by GAWLS for St, THFMA, CHMA, GMA, and PACS; method-wise predicted vs observed concentration plots for each monomer combination; prediction ability (*R*^2^) by univariate linear regression models using selected peaks; and prediction ability (*R*^2^) by multivariate linear regression models using a set of selected peaks (PDF)
IR spectral data sets of copolymerization reactions with monomer concentrations (XLSX)
