# Supplementary material for: Chemometrics Approach Based on Wavelet Transforms for the Estimation of Monomer Concentrations from FTIR Spectra
Source: ACS Omega. 2023 May 23;8(22):19781–8. doi: 10.1021/acsomega.3c01515 (PMC10249027; doi:10.1021/acsomega.3c01515)
Supplement: Supplementary file 1 — ao3c01515_si_001.pdf [file ao3c01515_si_001.pdf]

Supporting Information for

Chemometrics Approach based on Wavelet Transform for the Estimation of Monomer Concentrations from FTIR Spectra

Araki Wakiuchi[1,3], *Swarit Jasial*[2,3], *Shigehito Asano*[4], *Ryo Hashizume*[4], *Miho Hatanaka*[5], *Yu-ya Ohnishi*[1], *Takamitsu Matsubara*[3,2], *Hiroharu Ajiro*[3,2], *Tetsunori Sugawara*[4], *Mikiya Fujii*[3,2], *Tomoyuki Miyao*[2,3]*

[1]Materials Informatics Initiative, RD technology and digital transformation center, JSR Corporation, 3-103-9 Tonomachi, Kawasaki-ku, Kawasaki, Kanagawa, 210-0821, Japan.

[2]Data Science Center, Nara Institute of Science and Technology, 8916-5 Takayama-cho, Ikoma, Nara, 630-0192, Japan.

[3]Graduate School of Science and Technology, Nara Institute of Science and Technology, 8916-5 Takayama-cho, Ikoma, Nara, 630-0192, Japan.

[4] JSR Corporation Yokkaichi Research Center, 100 Kawajiri-cho, Yokkaichi, Mie, 510-8552, Japan.

[5]Department of Chemistry, Faculty of Science and Technology, Keio University, 3-14-1 Hiyoshi, Kohoku-ku, Yokohama, Kanagawa 223-8522, Japan.

*Corresponding author

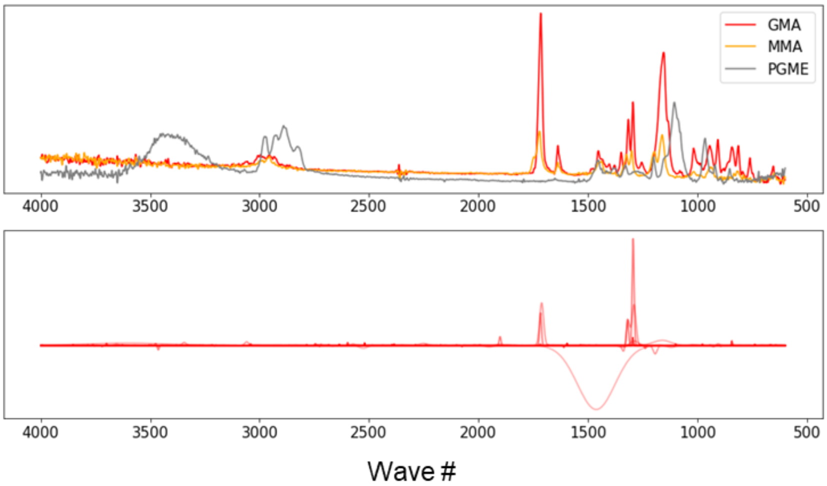

Figure S1. Interpretation of a WT10-ENCV Model for a GMA concentration prediction. FTIR spectra of monomers and PGME (top), and the visualization of contribution of peaks for the predicted concentration from the average of coefficients in all trained WT10-ENCVs with scaling obtained from five times of 5-fold cross-validation. (bottom).

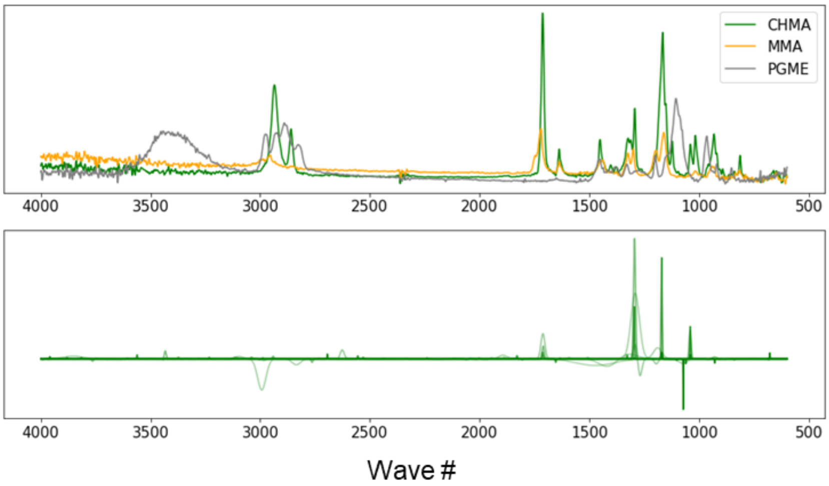

Figure S2. Interpretation of a WT10-ENCV Model for a CHMA concentration prediction. FTIR spectra of monomers and PGME (top), and the visualization of contribution of peaks for the predicted concentration from the average of coefficients in all trained WT10-ENCVs with scaling obtained from five times of 5-fold cross-validation. (bottom).

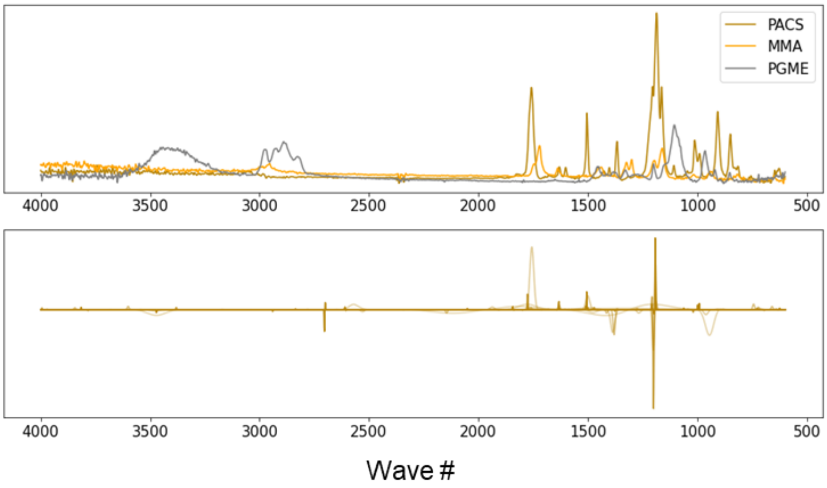

Figure S3. Interpretation of a WT10-ENCV Model for a PACS concentration prediction. FTIR spectra of monomers and PGME (top), and the visualization of contribution of peaks for the predicted concentration from the average of coefficients in all trained WT10-ENCVs with scaling obtained from five times of 5-fold cross-validation. (bottom).

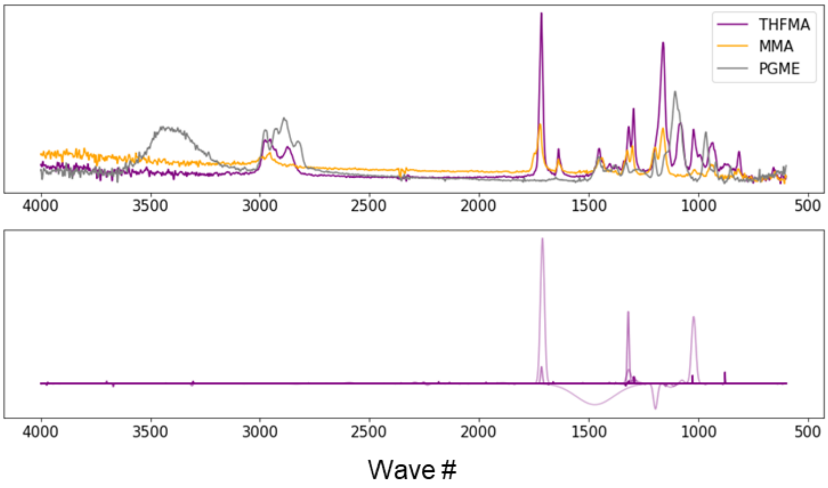

Figure S4. Interpretation of a WT10-ENCV Model for a THFMA concentration prediction. FTIR spectra of monomers and PGME (top), and the visualization of contribution of peaks for the predicted concentration from the average of coefficients in all trained WT10-ENCVs with scaling obtained from five times of 5-fold cross-validation. (bottom).

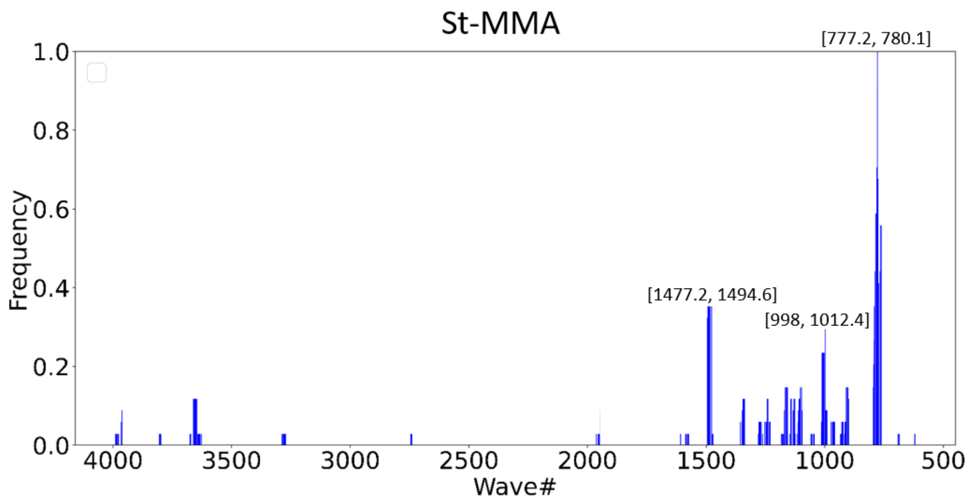

Figure S5. Important wavelength numbers/regions from GAWLS Model for St concentration prediction. The bar plot shows the frequency of occurrence (scaled) of each wave number/region. The height of the bar for a wavenumber represents its importance in terms of scaled frequency (y axis). Top wave numbers/regions based on frequency are annotated on their respective bars.

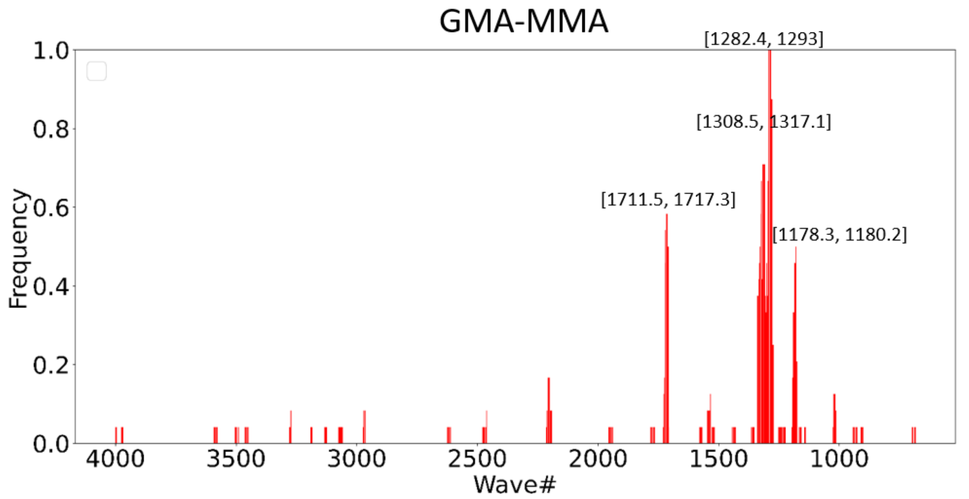

Figure S6. Important wavelength numbers/regions from GAWLS Model for GMA concentration prediction. The bar plot shows the frequency of occurrence (scaled) of each wave number/region. The height of the bar for a wavenumber represents its importance in terms of scaled frequency (y axis). Top wave numbers/regions based on frequency are annotated on their respective bars.

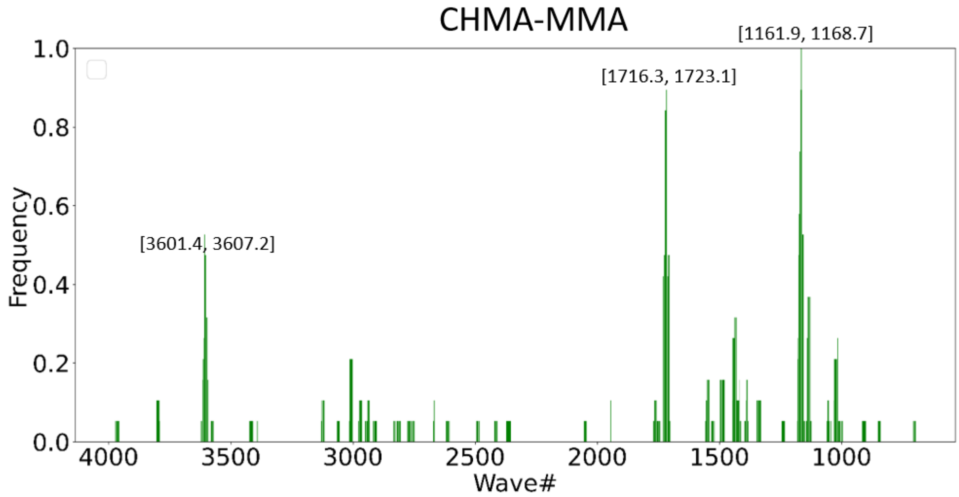

Figure S7. Important wavelength numbers/regions from GAWLS Model for CHMA concentration prediction. The bar plot shows the frequency of occurrence (scaled) of each wave number/region. The height of the bar for a wavenumber represents its importance in terms of scaled frequency (y axis). Top wave numbers/regions based on frequency are annotated on their respective bars.

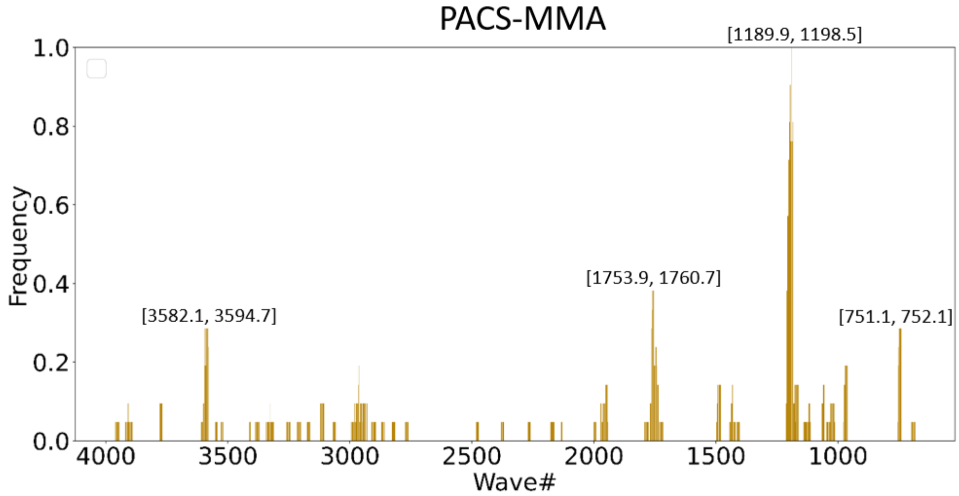

Figure S8. Important wavelength numbers/regions from GAWLS Model for PACS concentration prediction. The bar plot shows the frequency of occurrence (scaled) of each wave number/region. The height of the bar for a wavenumber represents its importance in terms of scaled frequency (y axis). Top wave numbers/regions based on frequency are annotated on their respective bars.

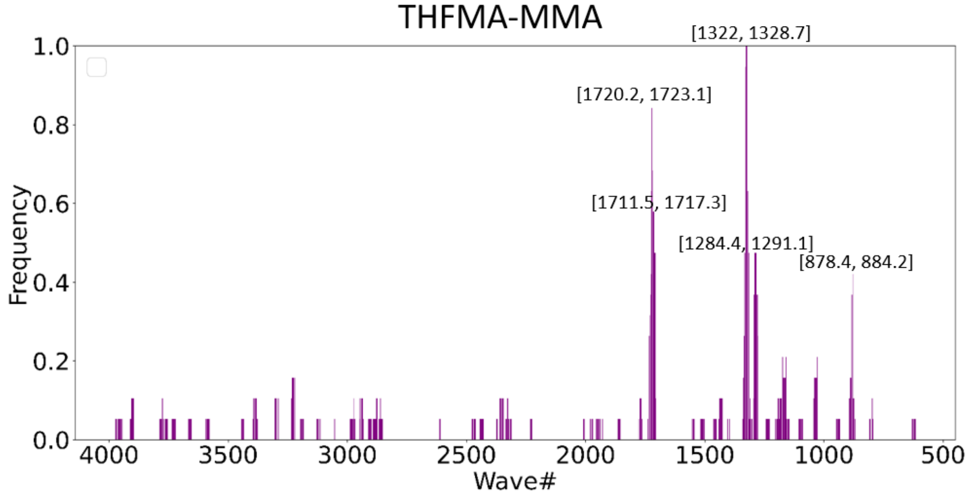

Figure S9. Important wavelength numbers/regions from GAWLS Model for THFMA concentration prediction. The bar plot shows the frequency of occurrence (scaled) of each wave number/region. The height of the bar for a wavenumber represents its importance in terms of scaled frequency (y axis). Top wave numbers/regions based on frequency are annotated on their respective bars.

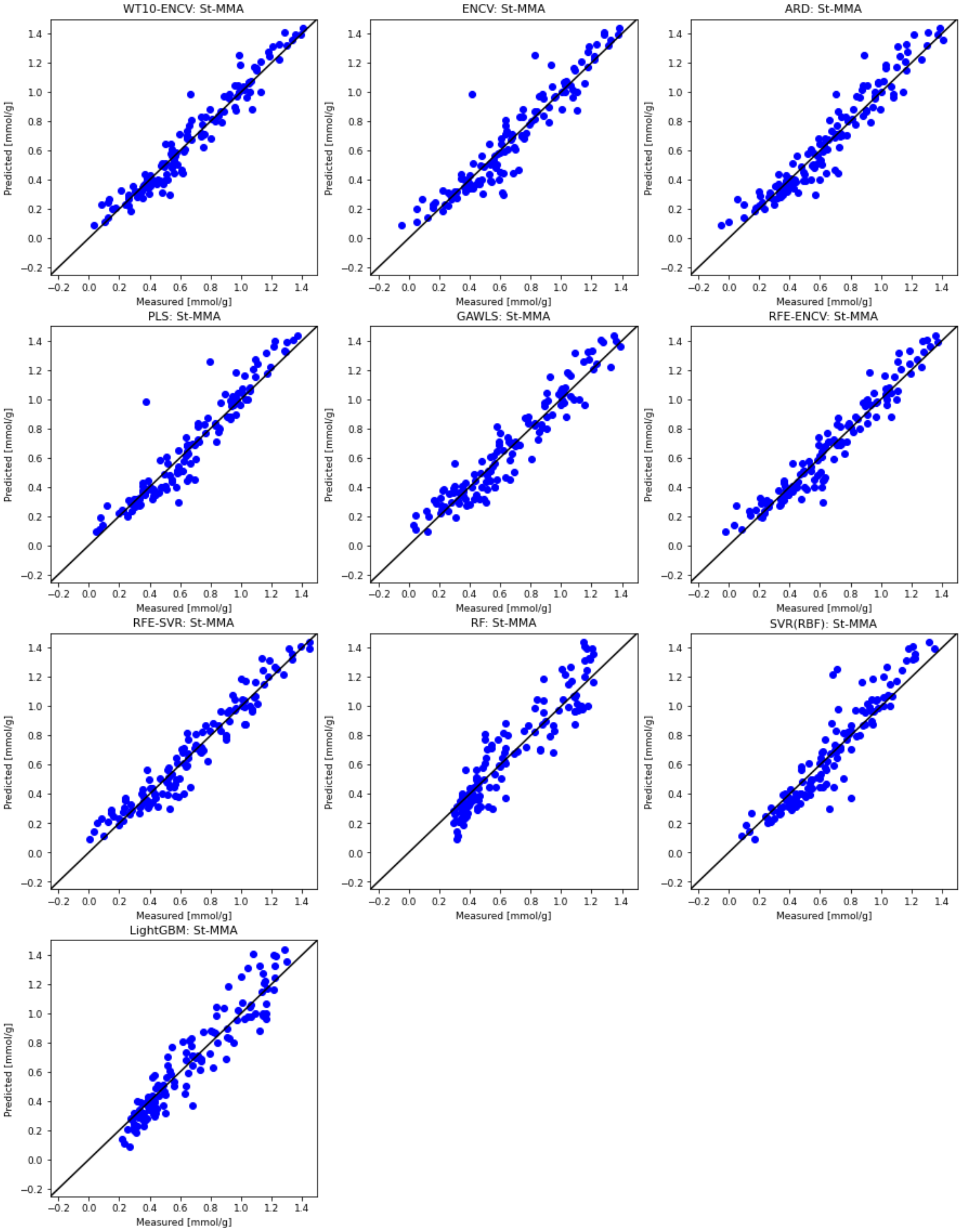

Figure S10. Examples of predicted vs measured concentration plots by the ML models in Table 2 for St and MMA by cross validation on the same random seed.

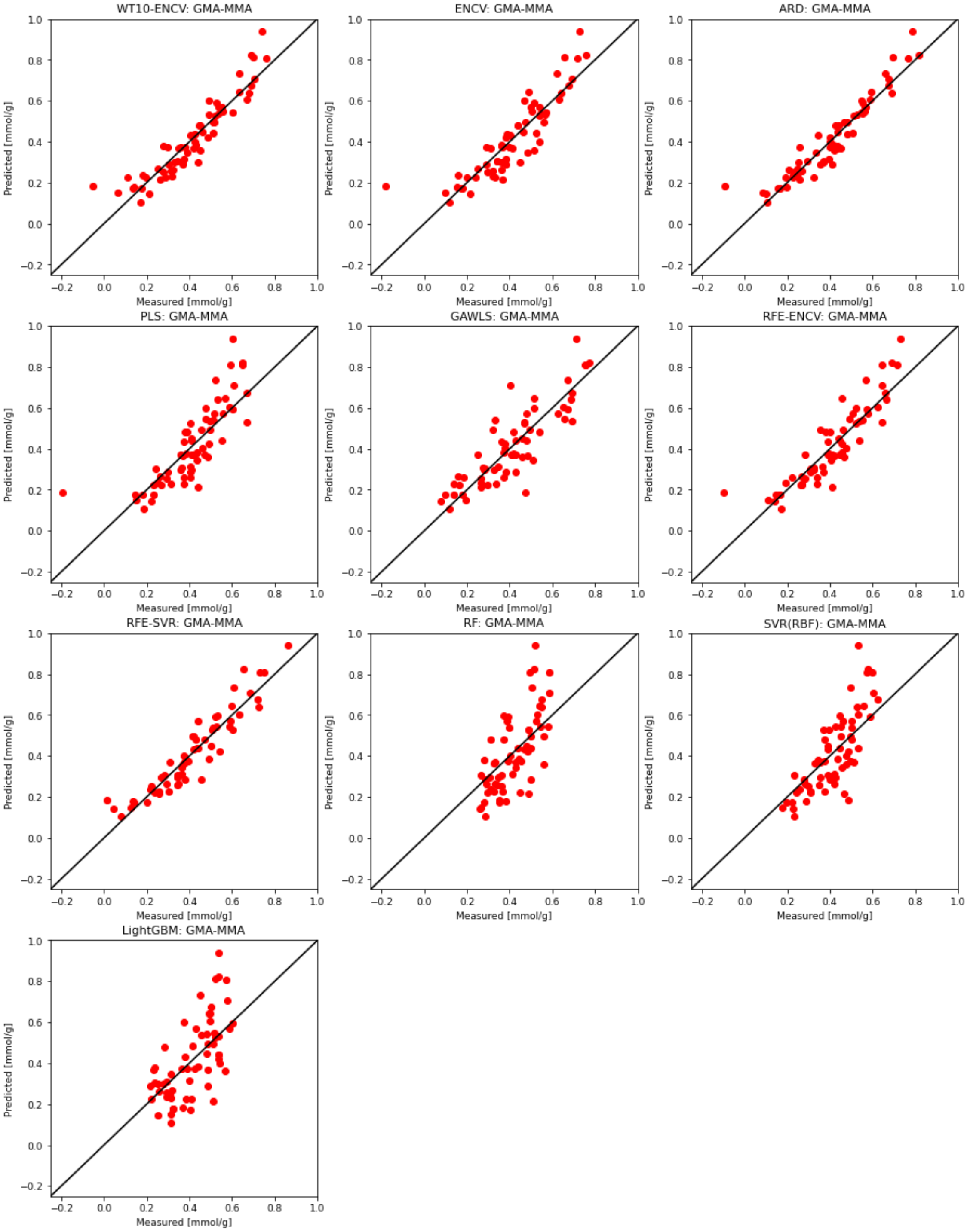

Figure S11. Examples of predicted vs measured concentration plots by the ML models in Table 2 for GMA and MMA by cross validation on the same random seed.

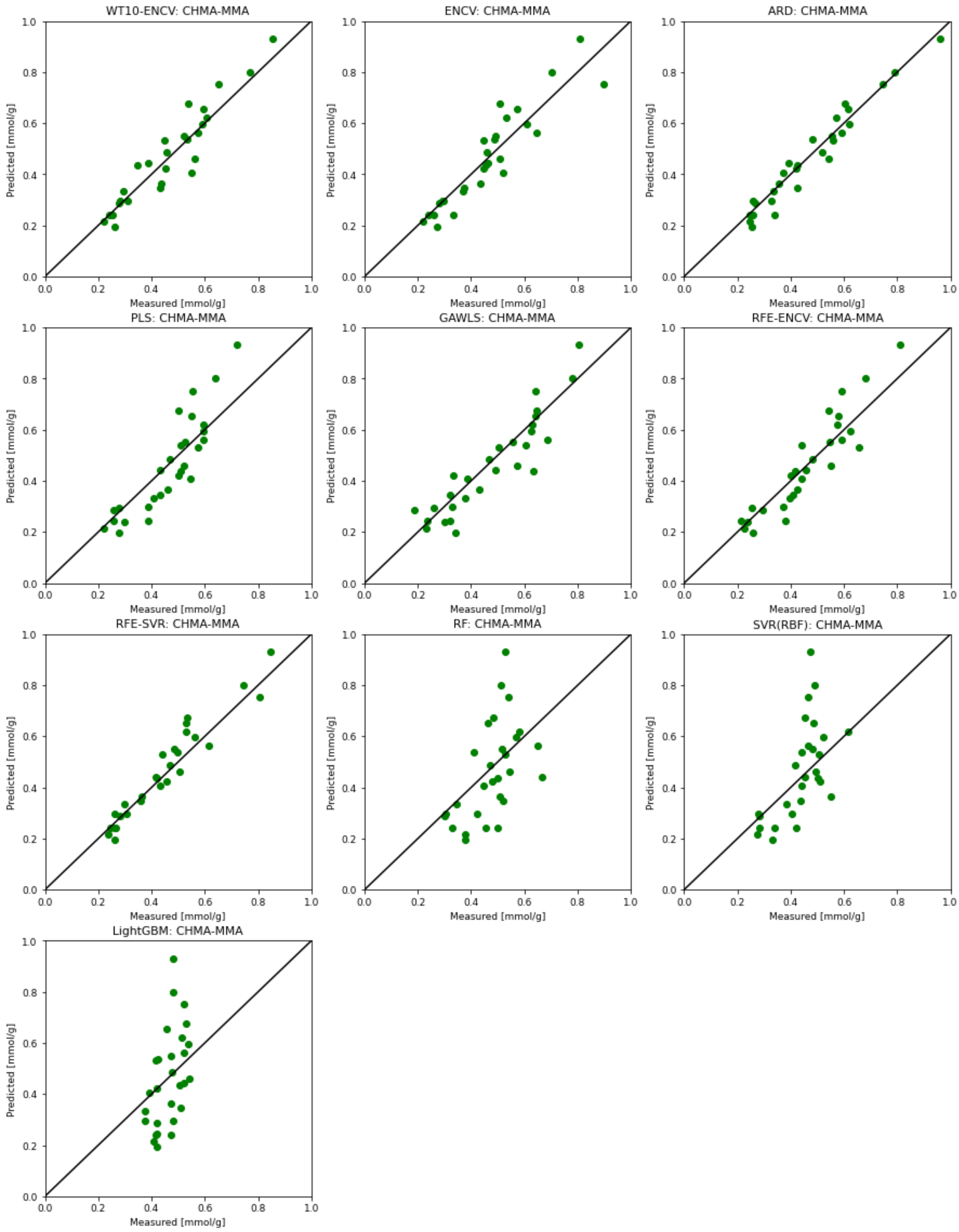

Figure S12. Examples of predicted vs measured concentration plots by the ML models in Table 2 for CHMA and MMA by cross validation on the same random seed.

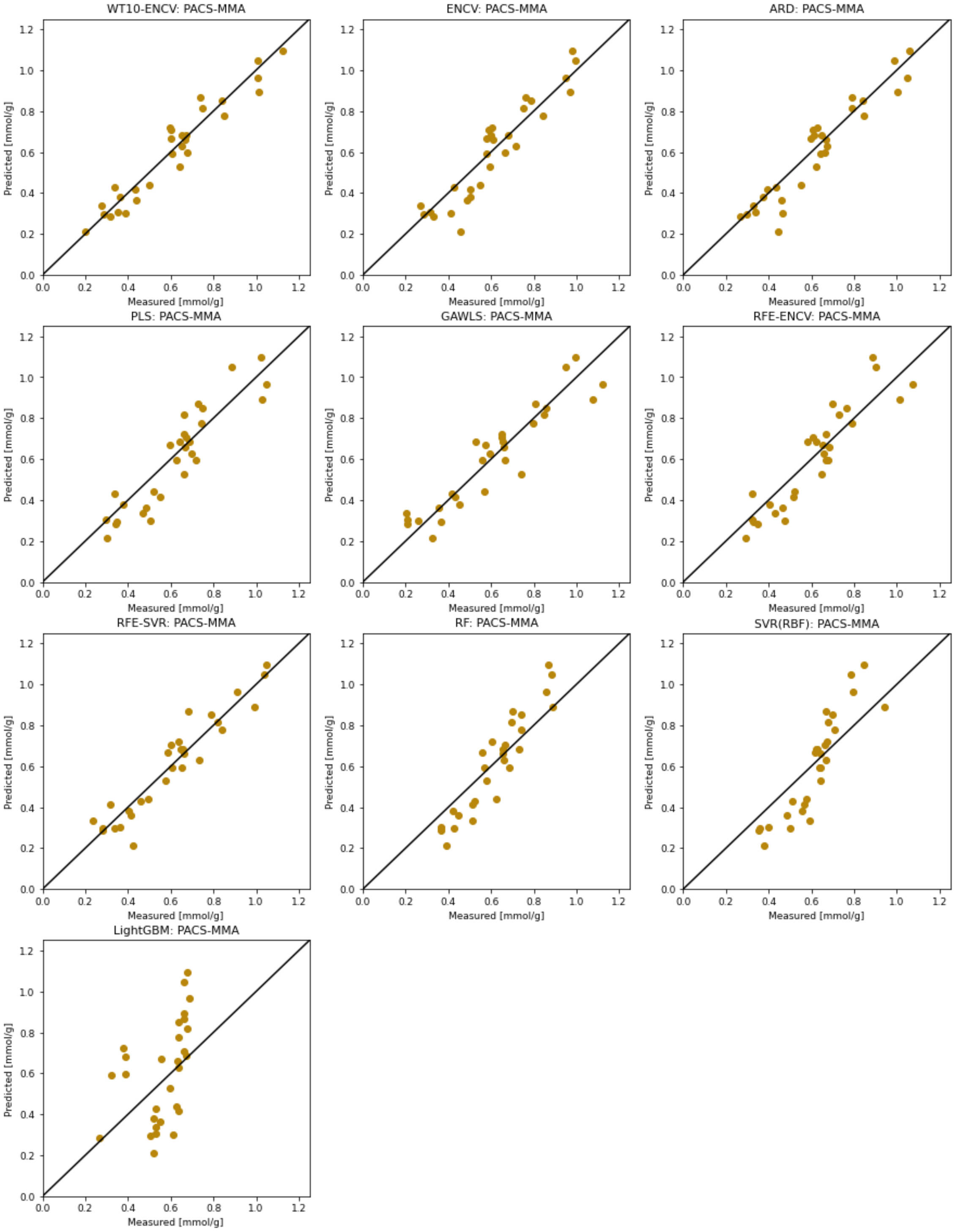

Figure S13. Examples of predicted vs measured concentration plots by the ML models in Table 2 for PACS and MMA by cross validation on the same random seed.

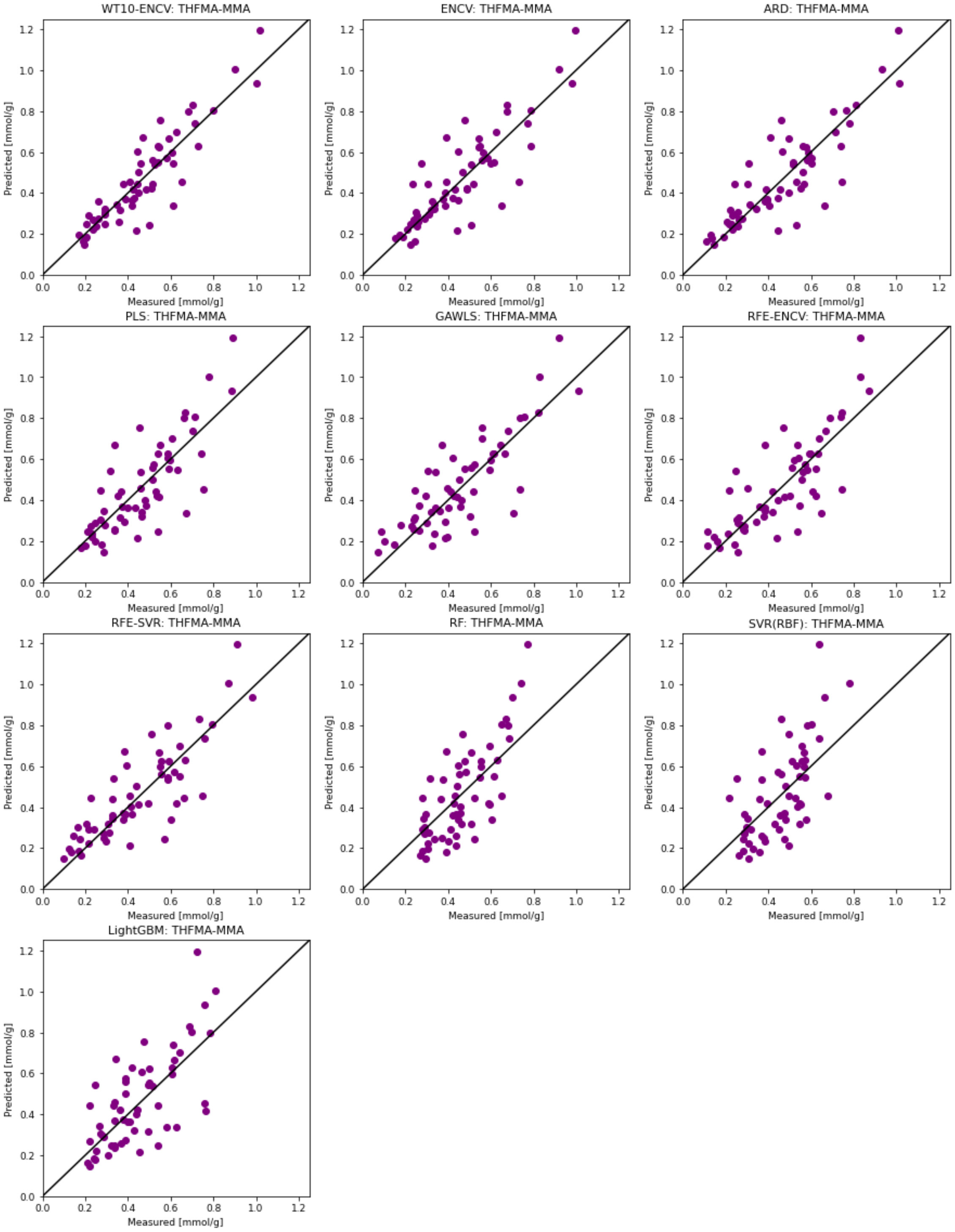

Figure S14. Examples of predicted vs measured concentration plots by the ML models in Table 2 for THFMA and MMA by cross validation on the same random seed.

Table S1. Linear regression for individual peaks (conventional method). Linear regression results are shown for the individual selected peaks from the pure spectra of each monomer.

St		GMA		CHMA		PACS		THFMA	
**Peaks**	R^2	**Peaks**	R^2	**Peaks**	R^2	**Peaks**	R^2	**Peaks**	R^2
**694.2**	0.29	**653.8**	-0.04	**1017.3**	0.38	**627.7**	-0.10	**1022.1**	0.61
**774.3**	0.68	**760.8**	-0.04	**1039.4**	0.32	**908.3**	0.57	**1080.9**	0.01
**906.4**	0.55	**907.3**	0.18	**1123.3**	-0.15	**1012.4**	0.75		
**990.3**	0.16	**1017.3**	0.11	**2859.9**	-0.15	**1367.3**	0.58		
**1493.6**	0.08	**1348**	0.001	**2935.1**	-0.17	**1505.2**	0.87		
**3026.7**	0.03	**2995.9**	-0.05			**1757.8**	0.72		
						**3744.1**	-0.15		

Table S2. Linear regression for combination of peaks (conventional method). Linear regression results are shown for the combination of selected peaks from the pure spectra of each monomer.

	Linear Regression
**St**	0.67
**GMA**	-0.08
**CHMA**	0.52
**PACS**	0.82
**THFMA**	0.66
